# A first-principles investigation of the linear thermal expansion coefficients of BeF_2_: giant thermal expansion

**DOI:** 10.1039/d2ra04860d

**Published:** 2022-09-20

**Authors:** Chee Kwan Gan, Abdullah I. Al-Sharif, Ammar Al-Shorman, Abdallah Qteish

**Affiliations:** Institute of High Performance Computing 1 Fusionopolis Way, #16-16 Connexis 138632 Singapore ganck@ihpc.a-star.edu.sg; Department of Physics, Yarmouk University Irbid-21163 Jordan aqteish@yu.edu.jo

## Abstract

We present the results of a theoretical investigation of the linear thermal expansion coefficients (TECs) of BeF_2_, within a direct Grüneisen formalism where symmetry-preserving deformations are employed. The required physical quantities such as the optimized crystal structures, elastic constants, mode Grüneisen parameters, and phonon density of states are calculated from first-principles. BeF_2_ shows an extensive polymorphism at low pressures, and the lowest energy phases [α-cristobalite with space group (SG) *P*4_1_2_1_2 and its similar phase with SG *P*4_3_2_1_2] are considered in addition to the experimentally observed α-quartz phase. For benchmarking purposes, similar calculations are performed for the rutile phase of ZnF_2_, where the volumetric TEC (*α*_v_), derived from the calculated linear TECs along the *a* (*α*_*a*_) and *c* (*α*_*c*_) directions, is in very good agreement with experimental data and previous theoretical results. For the considered phases of BeF_2_, we do not find any negative thermal expansion (NTE). However we observe diverse thermal properties for the distinct phases. The linear TECs are very large, especially *α*_*c*_ of the α-cristobalite phase and its similar phase, leading to giant *α*_v_ (∼175 × 10^−6^ K^−1^ at 300 K). The giant *α*_v_ arises from large Grüneisen parameters of low-frequency phonon modes, and the *C*_13_ elastic constant that is negatively signed and large in magnitude for the α-cristobalite phase. The elastic constants, high-frequency dielectric constants, Born effective charge tensors, and thermal properties of the above phases of BeF_2_ are reported for the first time and hence serve as predictions.

## Abstract 2

A first-principles investigation of the linear thermal expansion coefficients of BeF_2_.

## Graphical abstract

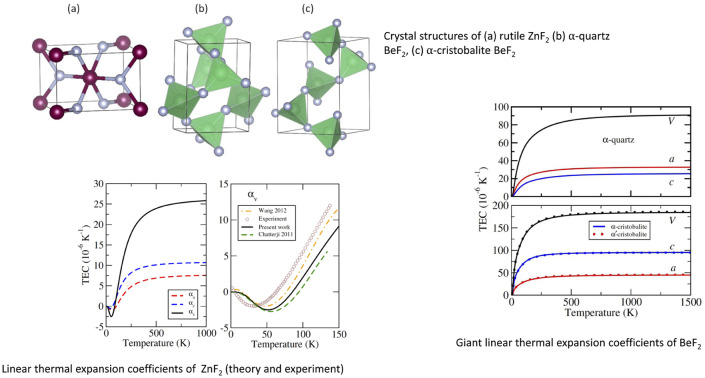

## 1. Introduction

Beryllium fluoride (BeF_2_) is known to exist in glass and crystalline phases and has a variety of technological applications. BeF_2_ glass has a large bandgap of about 13.8 eV, the lowest refractive index and highest Abbe number of any inorganic material, and exceptional resistance to damage. These properties have enabled the manufacturing of special glasses (from BeF_2_ and its mixtures with fluorides and other difluorides) that have excellent transmittance in the UV region^1,2^ and for use in high-power laser systems.^3^ The LiF–BeF_2_ mixture is a primary coolant and fuel solvent in molten salt nuclear reactors.^4^ In protein crystallography, BeF_2_ is used to restrict protein motion to facilitate the crystallography process.^5^ Very recently, crystalline BeF_2_ is predicted to be a better neutron filter than MgF_2_, which has been considered an effective neutron filter candidate.^6^ The main aim of this work is to investigate, for the first time, the linear thermal expansion coefficients (TECs) of a few low-energy crystalline phases of BeF_2_. We also consider a benchmark system ZnF_2_ that has exceptional electric and optical properties, and interesting technological applications ranging from catalysis to spectroscopy and laser applications.^7^

Single crystal BeF_2_ has been grown and found to have a crystal structure remarkably similar to that of the α-quartz (SiO_2_) structure,^8^ which has a trigonal symmetry with space group (SG) *P*3_1_21 (#152). A recent first-principles study has revealed that BeF_2_ shows extensive polymorphism at low pressures.^9^ Interestingly, three crystal phases [namely, (i) the α-cristobalite phase that has a tetragonal symmetry with SG *P*4_1_2_1_2 (#92), (ii) a similar phase to the α-cristobalite phase (hereafter referred to as the α′-cristobalite phase) with SG *P*4_3_2_1_2 (#96), and (iii) the *C*2/*c*-2 × 4 phase with SG *C*12/*c*1 (#15)] are predicted to be energetically more stable than α-quartz. However, these phases have a very small stability pressure range (less than 0.4 GPa), and the α-quartz phase transforms to the coesite-I phase SG *C*2/*c* at 3.1 GPa. The high-pressure phases of BeF_2_ have been the subject of other first-principles calculations.^10^ Very recently, first-principles calculations have also been employed to construct the *P*–*T* phase diagram of BeF_2_.^11^ The HSE06 optical bandgap of the α-quartz structure is found to be about 10.6 eV, and increases by increasing the applied pressure.^9^ The lattice vibrations, inelastic scattering cross-sections, and neutron transmission of BeF_2_ have been thoroughly investigated^6^ using first-principles calculations and compared to those of MgF_2_.

The benchmark system ZnF_2_ crystallizes in the tetragonal rutile structure with SG *P*4_2_/*mnm* (#136). Very recently, Raman scattering measurements with the use of the diamond anvil cell have been employed to investigate the structural phase transformations of ZnF_2_ under high pressures.^12^ This experimental work is supplemented by first-principles calculations. In addition to the structural stability and pressure variation of the Raman active phonon modes, the electronic bandgap of the considered phases as a function of pressure has been investigated at the HSE06 level. Neutron diffraction has been employed to study the temperature dependence of the lattice parameters and unit cell volume of ZnF_2_,^13^ and NTE has been observed in a small temperature range (below 75 K). This NTE behavior has been supported by first-principles calculations.^13,14^ However, only the volumetric TEC has been theoretically investigated.

In the present work, the linear TECs of BeF_2_ and ZnF_2_ are investigated by employing the recently introduced direct approach^15^ in which the symmetry of the deformed structures could be preserved. Since this approach has not been applied to systems with tetragonal symmetry, ZnF_2_ is thus chosen as a suitable benchmark system because of its tetragonal crystal structure, in addition to the existence of experimental and previous theoretical results of its volumetric thermal expansion. The elastic constants and phonon frequencies required to compute linear TECs are calculated from first-principles. For BeF_2_, the α-quartz, α-cristobalite and α′-cristobalite phases will be considered. Moreover, the relative stability of the above three phases of BeF_2_ are also investigated using different levels of approximation of the exchange-correlation potential.

## 2. Methodology

The linear TECs of the considered phases of ZnF_2_ and BeF_2_ are calculated within the Grüneisen formalism following the procedure described in ref. 16–22. To compute the mode Grüneisen parameters we considered two types of symmetry-preserving deformations obtained by changing the in-plane (*a*) or out-of-plane (*c*) lattice parameters by ±0.5%. These deformations allow for the full utilization of the tetragonal or trigonal point-group symmetry^23^ of the considered systems, which minimizes the required number of independent atomic displacements (*i.e.*, number of supercells) to calculate the phonon frequencies within the direct method.^24–28^ The amplitude of atomic displacements, from the corresponding equilibrium positions, is 0.015 Å. The supercell sizes are of 2 × 2 × 3 for ZnF_2_, and 2 × 2 × 2 for the α-quartz, α-cristobalite, and α′-cristobalite phases of BeF_2_. The adequacy of these supercells have been checked by considering larger ones for each of these systems, and we found that such actions do not alter appreciably our main results and conclusions. The determination of the linear TECs also requires the elastic constants that may be obtained through fittings of energy *versus* strain curves.^29,30^ Specifically, these TECs at temperature *T* in the *a* (*α*_*a*_) and *c* (*α*_*c*_) directions of the above systems are given by1
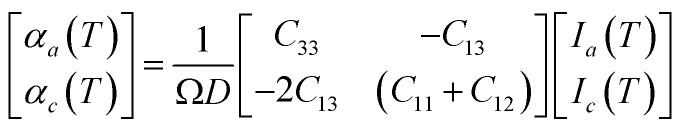
where *C*_*ij*_ are the elastic constants, *D* = (*C*_11_ + *C*_12_)*C*_33_ − 2*C*_13_^2^ and *Ω* is the volume of the primitive cell. The phonon density of states (PDOS) weighted by the Grüneisen parameters are2
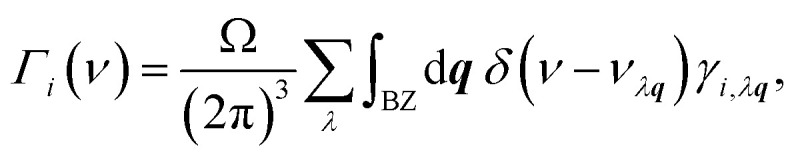
with the Grüneisen parameter *γ*_*i*,*λ****q***_ = −*ν*_*λ****q***_^−1^∂*ν*_*λ****q***_/∂*ε*_*i*_ for the deformation of type *i* (with *i* = *a* for in-plane and *i* = *c* for out-of-plane deformations). The derivative ∂*ν*_*λ****q***_/∂*ε*_*i*_ measures the change of the frequency *ν*_*λ****q***_ with respect to the strain parameter *ε*_*i*_.^15^ The summation is over all frequencies *ν*_*λ****q***_ for the phonon band index *λ* and ***q*** vector in the Brillouin zone (BZ). The *I*_*i*_(*T*) are calculated from3
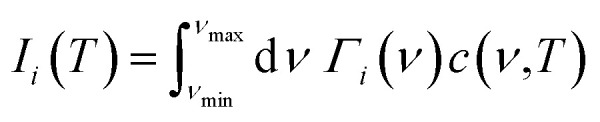
where *c*(*ν*,*T*) = *k*_B_(*r*/sinh *r*)^2^ is the contribution of the phonon modes with frequency *ν* to the specific heat. Here, *r* = *hν*/2*k*_B_*T*, and *h* and *k*_B_ are the Planck and Boltzmann constants, respectively. The maximum (minimum) frequency is denoted by *ν*_max_ (*ν*_min_).

The DFT calculations of the optimized structural parameters, phonon frequencies, and elastic constants are performed by employing the projector augmented wave (PAW) method, as implemented in the Vienna *Ab Initio* Simulation Package (VASP). A relatively high cutoff energy of 600 eV is used for the plane-wave basis. Geometry optimization is stopped when the maximum force on each atom is less than 10^−3^ eV Å^−1^. We find that phonon frequency shifts are more consistent when we use the local density approximation (LDA) for BeF_2_, and the PBE_sol functional of the generalized gradient approximation (GGA) for ZnF_2_. Therefore, for the linear TECs only the results of these calculations are reported.

## 3. Results and discussion

### 3.1. Structural properties

**Fig. 1:**
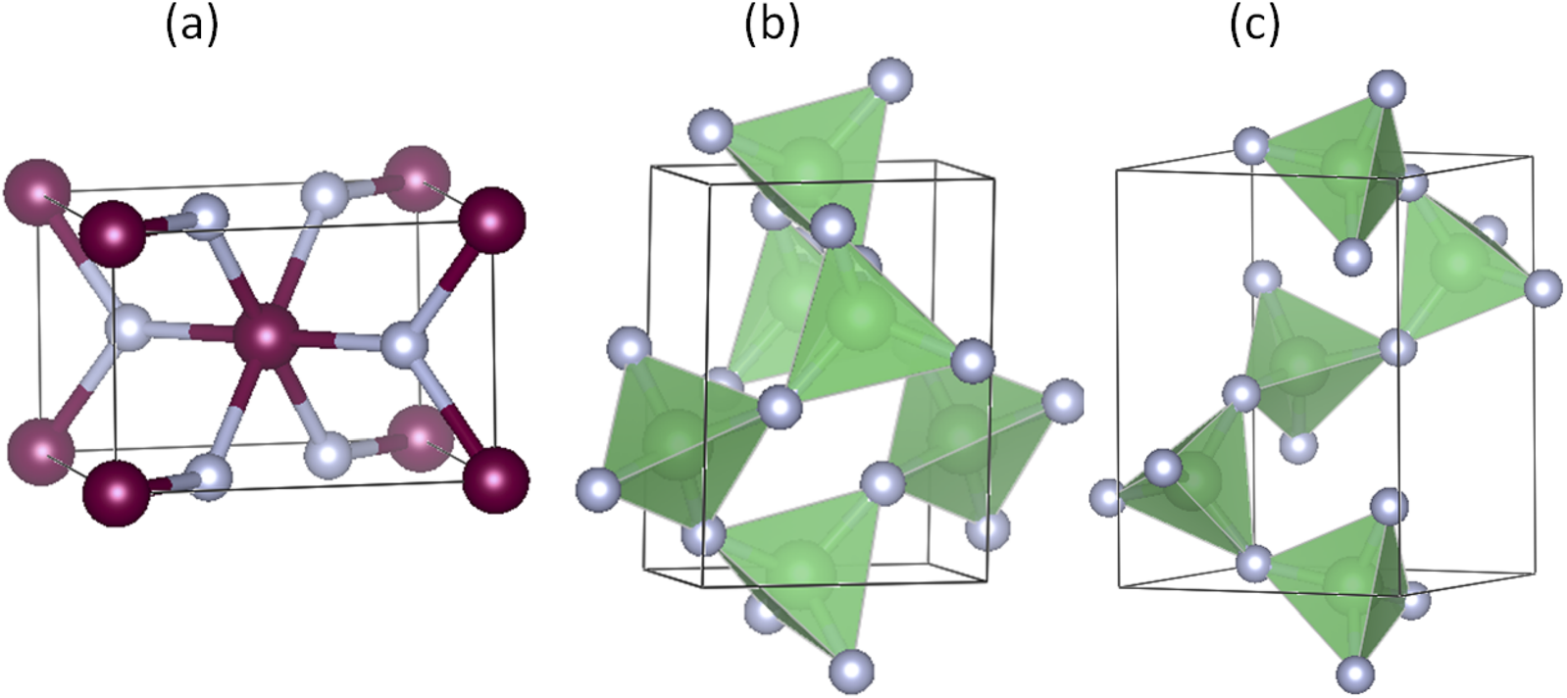
The crystal structures of (a) rutile ZnF_2_, (b) α-quartz BeF_2_, and (c) α-cristobalite BeF_2_. The α′-cristobalite BeF_2_ structure which is similar to the α-cristobalite BeF_2_ structure is not shown. The small gray balls represent the F atoms. The *c* axis of the three crystal structures is along the vertical direction.

We start with the benchmark system, ZnF_2_, which at ambient conditions crystallizes in the rutile structure, shown in Fig. 1(a). This crystal structure has a tetragonal symmetry and six atoms per primitive unit cell. The two Zn atoms occupy the Wyckoff 2a(0, 0, 0) sites and the four F atoms are located at the 4f(*x*, *x*, 0) sites. Therefore, this structure is characterized by three crystallographic parameters: two lattice parameters (*a* and *c*) and an internal parameter for the coordinates of the four F atoms (*x*). The structural parameters obtained using the PBE_sol functional are (*a*, *c*, *x*) = (4.7194 Å, 3.1376 Å, 0.3037), while the corresponding LDA results are (4.6373 Å, 3.0990 Å, 0.3033). These calculated values are in very good agreement with the experimental data (4.7038 Å, 3.1336 Å, 0.3035)^32^ and (4.7034 Å, 3.1335 Å, 0.303).^13^ As expected, the LDA lattice parameters are underestimated while those of the PBE_sol are slightly overestimated.

For BeF_2_, the three considered crystal structures are α-quartz [Fig. 1(b)], α-cristobalite [Fig. 1(c)] and α′-cristobalite. The α-quartz phase has trigonal symmetry and nine atoms in the conventional hexagonal unit cell. The three Be atoms occupy the Wyckoff 3a(*x*_1_, *x*_1_, 0) sites and the six F atoms occupy the 6c(*x*_2_, *y*_2_, *z*_2_) sites. On the other hand, the α-cristobalite phase has a tetragonal symmetry and twelve atoms per primitive unit cell. The four Be atoms occupy the Wyckoff 4a(*x*_1_, *x*_1_, 0) sites and the eight F atoms occupy the 8c(*x*_2_, *y*_2_, *z*_2_) sites. Therefore, each of these two structures has six crystallographic parameters: two lattice parameters (*a* and *c*), and four internal parameters (denoted as *x*_1_, *x*_2_, *y*_2_ and *z*_2_). The atomic coordinates of the α′-cristobalite phase can be obtained from those of the α-cristobalite by mirror-image transformation (*x*, *y*, *z*) → (−*y*, −*x*, *z*), and the lattice parameters of the two structures are identical. Therefore, only the structure parameters of the first two crystal structures are reported. Our LDA, PBE_sol, and PBE results, shown in Table 1, are in good agreement with available experimental data and other theoretical calculations. The PBE_sol results lie between the corresponding LDA and PBE results and show the best agreement with the experimental data for the α-quartz.

**Table 1:** Calculated lattice constants and internal parameters of the α-quartz and α-cristobalite phases of BeF_2_. Also shown are the relative energy (Δ*E*) of the α-cristobalite phase with respect to that of α-quartz, and the available experimental data (measured at 100 K) and other theoretical results

Phase	Approach	Lattice constants		Internal parameters				Δ*E* (meV)
		*a* (Å)	*c* (Å)	*x*_1_	*x*_2_	*y*_2_	*z*_2_	
α-Quartz	LDA	4.5958	5.0529	0.4579	0.4098	0.2867	0.2290	
	PBE_sol	4.7301	5.1814	0.4662	0.4138	0.2737	0.2188	
	PBE	4.8497	5.3070	0.4756	0.4176	0.2575	0.2053	
	PBE^6^	4.8282	5.2837	0.4740	0.4171	0.2601	0.2075	
	LDA^31^	4.6663	5.1608					
	Expt.^8^	4.7390	5.1875	0.4700	0.4164	0.2671	0.2131	
α-Cristobalite	LDA	4.5967	6.1773	0.3226	0.2230	0.1454	0.2000	25
	PBE_sol	4.8087	6.5984	0.3044	0.2378	0.1112	0.1825	−2
	PBE	4.8934	6.7428	0.2988	0.2400	0.1001	0.1769	−9
	LDA^31^	4.695	6.318					
	LDA^11^	4.684	6.373					
	PBE^11^	4.960	6.910					

### 3.2. Elastic properties and stability

We show in Table 1 the LDA, PBE_sol and PBE relative energies (Δ*E*) of the α-cristobalite phase with respect to those of α-quartz. According to the PBE and PBE_sol calculations the latter phase is slightly more stable, in accordance with the Nelson *et al.*^9^ GGA calculations. However, the LDA calculations lead to an opposite conclusion. Similar conclusions have recently been reached by Masoumi,^11^ using both LDA and GGA calculations. These results show that these two phases have extremely close cohesive energies.

The α-quartz phase of BeF_2_ with a trigonal crystal symmetry has six independent elastic constants.^34^ On the other hand, the rutile phase of ZnF_2_ and α-cristobalite phase of BeF_2_ (both have a tetragonal crystal symmetry) also have six independent elastic constants.^34^ The elastic constants of these phases, obtained by using the LDA and PBE_sol functionals are shown in Table 2. There are two features to note from this table. First, our results for the rutile ZnF_2_ are in very good agreement with the available experimental values.^33^ Secondly, the PBE_sol values are systematically smaller than the corresponding LDA values, which is expected since the PBE_sol GGA functional leads to softer materials than LDA (see above). The calculated elastic constants are used in the calculations of the linear TECs (see Sec. 2).

**Table 2:** Calculated elastic constants of the α-quartz and α-cristobalite phases of BeF_2_, and the rutile phase of ZnF_2_

System	Phase	Approach	Elastic constants (GPa)						
			*C*_11_	*C*_12_	*C*_13_	*C*_14_	*C*_33_	*C*_44_	*C*_66_
BeF_2_	α-Quartz	LDA	46.975	14.223	12.067	−6.401	75.287	31.745	
		PBE_sol	42.278	4.991	3.760	−9.077	53.009	30.548	
	α-Cristobalite	LDA	33.309	7.300	−5.087		22.487	35.810	13.731
		PBE_sol	32.589	4.839	−5.336		24.412	37.813	16.078
ZnF_2_	Rutile	LDA	139.442	121.550	109.127		220.673	36.583	91.826
		PBE_sol	128.470	98.717	94.547		200.902	35.523	83.001
		Expt.^33^	125.5	91.8	83.0		192.2	39.5	80.7

The elastic constants could be used to investigate the mechanical stability of the crystal structure. For α-quartz structure, the Born stability criteria^34^ are

4 *D* = (*C*_11_ + *C*_12_)*C*_33_ − 2*C*_13_^2^ > 0,

and

5 (*C*_11_ − *C*_12_)*C*_44_ − 2*C*_14_^2^ > 0.

Note that the same expression of *D* appears in eqn (1). As for the rutile and α-cristobalite phases (of tetragonal (I) class),^35^ the Born stability criteria are: *C*_11_ > |*C*_12_|, *D* > 0, *C*_44_ > 0 and *C*_66_ > 0. The elastic constants reported in Table 2 show that these criteria are satisfied, and therefore the considered phases of ZnF_2_ and BeF_2_ are mechanically stable. The mechanical stability of these crystal structures can also be inferred from phonon dispersion relations, discussed below.

### 3.3. Phonon dispersion relations

Since the considered crystals are polar in character we perform non-analytical correction (NAC) to their dynamical matrices. To do that, we have calculated the high-frequency dielectric constant and Born effective charge tensors, and the results are listed in Table 3. The features to note from this table are the following. (i) The calculated results have a very weak dependence on the used exchange-correlation functional. (ii) The effective charges in the ZnF_2_ are larger than in BeF_2_, which shows that the ionicity of Zn–F bond is larger than that in Be–F. This is consistent with the corrected Allred–Rochow electronegativity values^39^ (larger for Be). (iii) Our calculated *xx* and *yy* components of the dielectric constant are in very good agreement with available experimental data,^36^ while that of *zz* is larger than the measured one. However, the comparable values of diagonal components of the dielectric constant in our calculations are consistent with the experimental and calculated values for other metal fluorides crystallizing in the rutile structure (such as MgF_2_ and FeF_2_).^40^

**Table 3:** Calculated high-frequency dielectric constants (DCs) and Born effective charges of the α-quartz (AQ) and α-cristobalite (AC) phases of BeF_2_, and the rutile phase of ZnF_2_

System	Phase	Approach	DC		Atom	Born effective charge								
			*xx* = *yy*	*zz*		*xx*	*xy*	*xz*	*yx*	*yy*	*yz*	*zx*	*zy*	*zz*
BeF_2_	AQ	PBE_sol	1.902	1.912	Be	1.728	0.000	0.000	0.000	1.914	0.081	0.000	−0.079	1.866
					F	−0.747	0.221	−0.115	0.213	−1.074	0.337	−0.095	0.345	−0.933
		LDA	1.887	1.896	Be	1.727	0.000	0.000	0.000	1.918	0.080	0.000	−0.0758	1.864
					F	−0.746	0.226	−0.124	0.218	−1.076	0.343	−0.104	0.356	−0.932
BeF_2_	AC	PBE_sol	1.723	1.718	Be	1.832	0.005	−0.045	0.005	1.832	0.049	0.100	−0.101	1.810
					F	−1.221	−0.117	0.377	−0.101	−0.611	0.061	0.380	0.100	−0.905
		LDA	1.827	1.818	Be	1.823	0.005	− 0.036	0.005	1.823	0.036	0.110	−0.110	1.790
					F	−1.199	−0.135	0.329	−0.120	−0.624	0.076	0.333	0.115	−0.896
ZnF_2_	Rutile	PBE_sol	2.549	2.664	Zn	2.222	−0.162	0.000	−0.162	2.222	0.00	0.000	0.000	2.424
					F	−1.111	−0.409	0.000	−0.409	−1.111	0.000	0.000	0.000	−1.200
		LDA	2.547	2.653	Zn	2.206	−0.1493	0.000	−0.1493	2.206	0.000	0.000	0.000	2.392
					F	−1.103	−0.395	0.000	−0.395	−1.103	0.000	0.000	0.000	−1.196
		Expt.^36^	2.6	2.1										

**Fig. 2:**
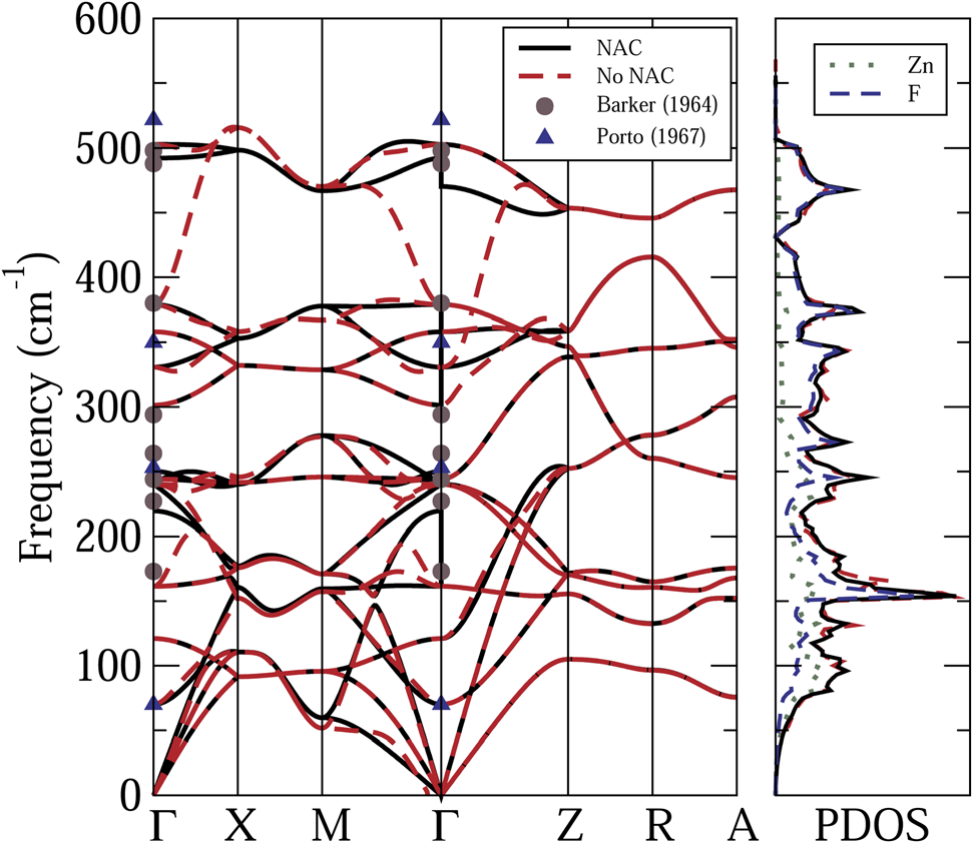
Calculated phonon dispersion relations and PDOS of ZnF_2_, with (black solid curves) and without (red dashed curves) non-analytic correction (NAC). The Zn and F projected PDOS, with NAC, and also shown. Symbols: available experimental data.^37,38^

Fig. 2 shows the calculated phonon dispersion relations and PDOS of the rutile ZnF_2_, with and without NAC. Also shown are the calculated Zn and F projected PDOS, with NAC, and the available experimental data.^37,38^ The frequency spans across an interval of about 500 cm^−1^. The features to note from this figure are the following. (i) As expected, the NAC leads to longitudinal optical-traverse optical (LO-TO) splitting, near the *Γ* point. The strongest effects are felt by high-frequency optical modes. However, the effects of the NAC on the calculated PDOS are quite small. (ii) Experimental data are available only for infrared^37^ and Raman^12,38^ active modes at the *Γ*-points. The reported frequencies of the latter modes are in very good agreement with each other, and hence only those of ref. 38 are shown in Fig. 2. For the designation of these phonon modes see ref. 40. Fig. 2 shows that these experimental data agree reasonably well with our first-principles results.

**Fig. 3:**
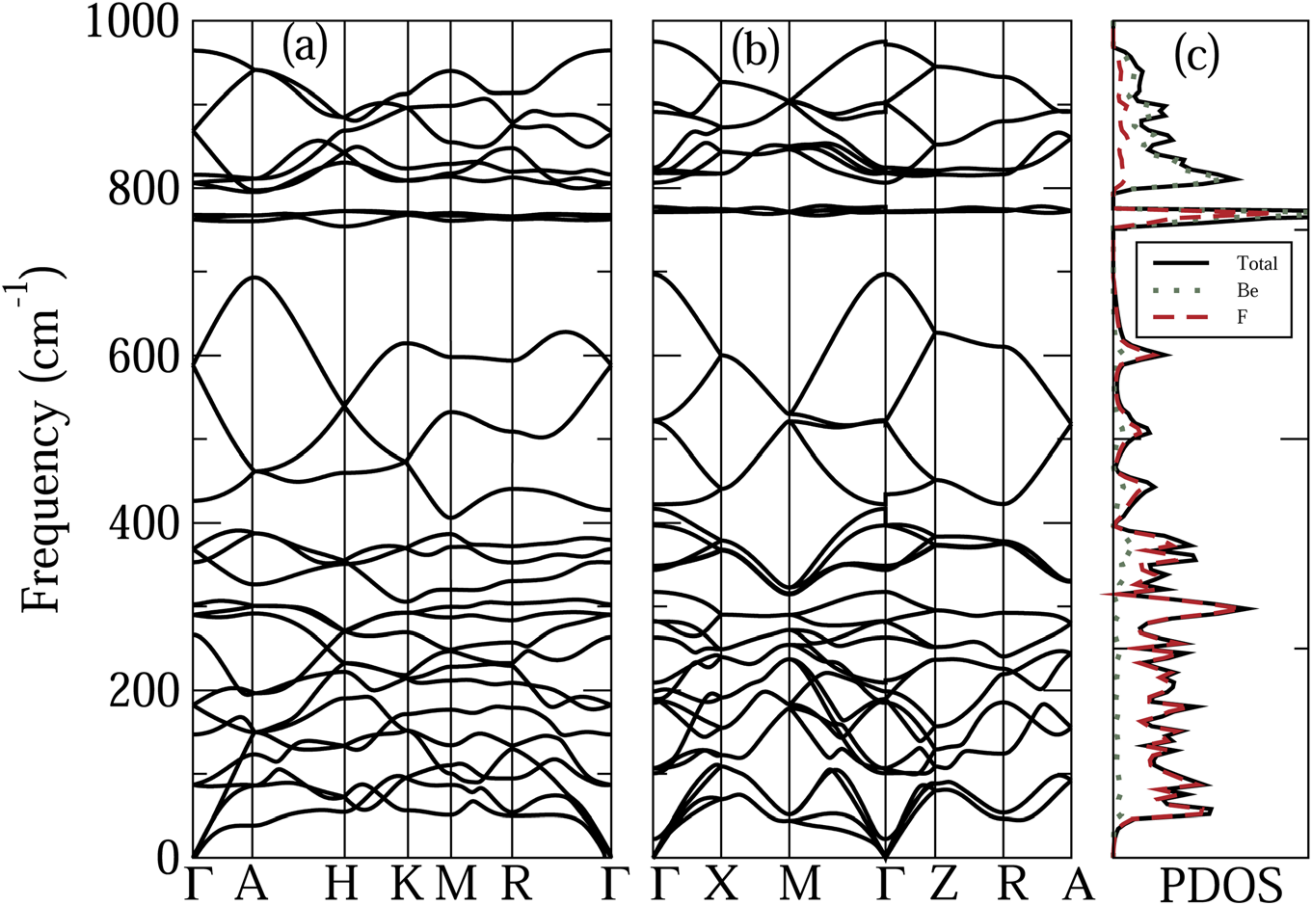
Calculated phonon dispersion relations of the (a) α-quartz and (b) α-cristobalite phases of BeF_2_. (c) PDOS, and the Be and F projected PDOS of the α-cristobalite phase.

Fig. 3 shows the phonon dispersion relations of the α-quartz and α-cristobalite phases of BeF_2_, taking into account the NAC. Also shown are the PDOS, and Be and F projected PDOS of the α-cristobalite phase. The results of the α′-cristobalite phase are very similar to those of α-cristobalite and hence are not shown. The features to note from this figure are the following. (i) The very wide frequency range of the phonon modes in these systems, compared to that of ZnF_2_. This can be understood as a consequence of the rather large mass difference between Be and Zn atoms. (ii) The frequency range of both phases of BeF_2_ can be separated, according to the character of the phonon modes, into three sub-regions. (a) The lower frequency region between 0 and about 700 cm^−1^, where the phonon modes are mainly due to the vibrations of F atoms. The contribution of the Be atoms becomes appreciable above 300 cm^−1^. It is worth noting that in the case of ZnF_2_ the dominance of the vibrations of the F atoms occurs in the upper part of the frequency range because the Zn atom is heavier than the F atom. (b) A narrow intermediate region at about 770 cm^−1^, where the rather localized phonon modes originate from vibrations involving both Be and F atoms. (c) The upper-frequency region, where the phonon modes originate mainly from vibrations of Be atoms. (iii) The opening of two frequency gaps, between (a) and (b), and between (b) and (c) sub-regions. These frequency gaps can be understood as a consequence of the localization of phonon modes in the (b) sub-region.

### 3.4. Thermal expansion

**Fig. 4:**
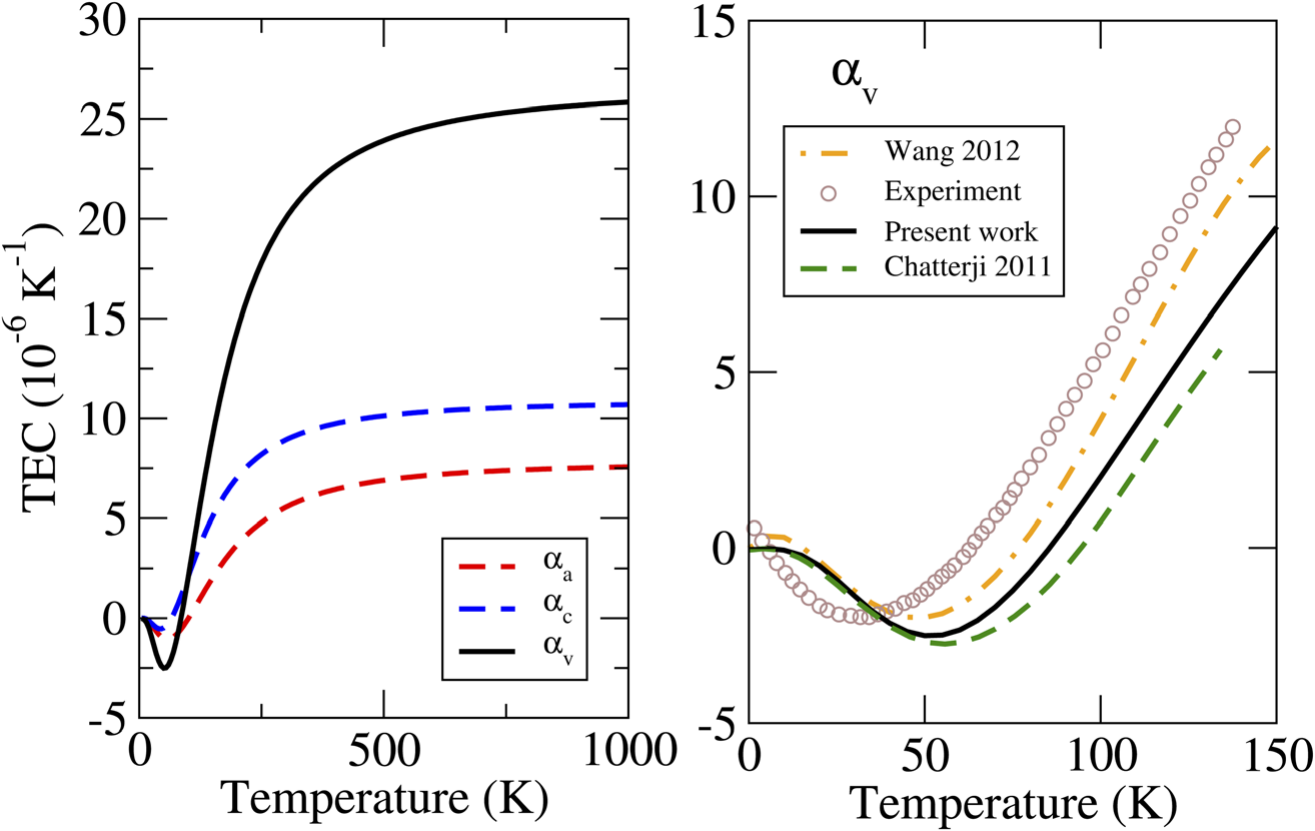
Calculated linear and volumetric TECs of ZnF_2_ using the LDA, compared to the available experimental data^13^ and previous theoretical results.^13,14^

**Fig. 5:**
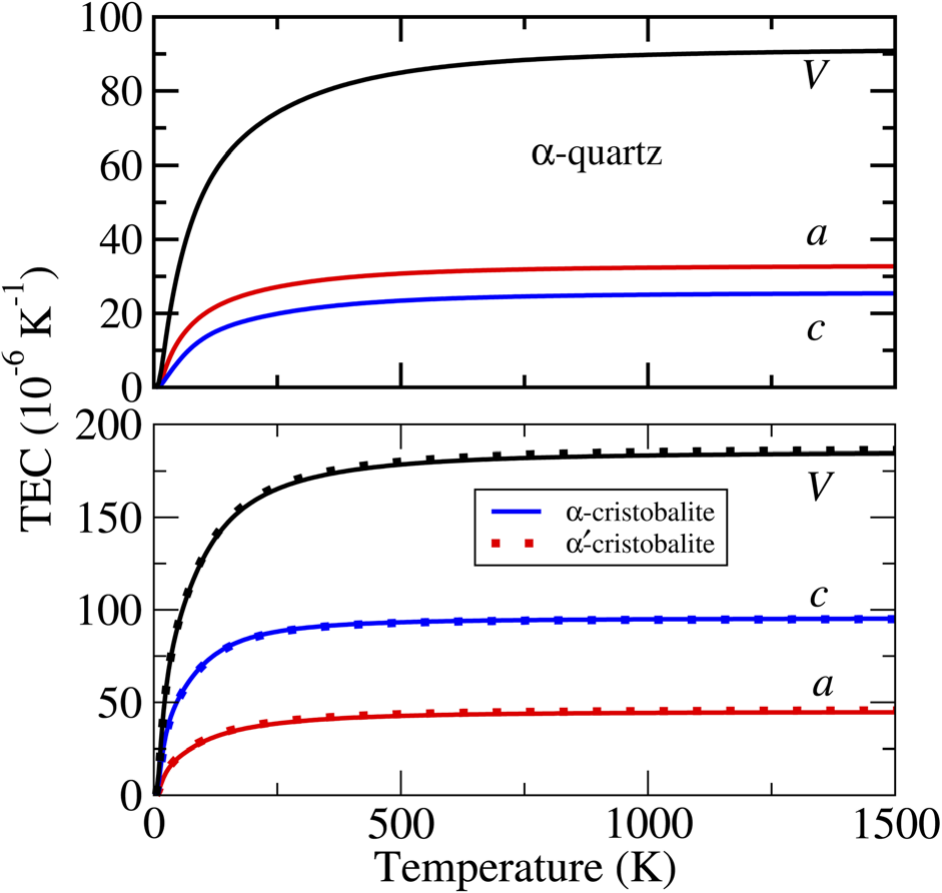
Calculated linear and volumetric TECs with PBE_sol of the three considered phases of BeF_2_. Note the difference in the scales of the two panels.

The calculated linear and volumetric TECs of the considered phases of ZnF_2_ and BeF_2_, according to the procedure described in Sec. 2, are depicted in Fig. 4 and 5, respectively. It is worth mentioning that, as expected, the NACs to the dynamical matrices have negligible effects on the calculated linear TECs.

We will first look at the TECs of ZnF_2_. The important features to note from Fig. 4 are the following. (i) The NTE at low temperatures is mostly due to *α*_*a*_. The negative values of *α*_*c*_ are smaller (in magnitude) than those of *α*_*a*_ and lie in a considerably shorter *T*-range. This is clear from the magnitude and the location of the minimum values: *α*_*a*_ ∼ −1.05 × 10^−6^ K^−1^ at 55 K, and *α*_*c*_ ∼ −0.5 × 10^−6^ K^−1^ at 40 K. These results are consistent with observed *T*-variations of the *a* and *c* lattice parameters at low temperatures (see Fig. 3 of ref. 13). (ii) The calculated *α*_v_ from the linear TECs (*i.e.*, *α*_v_ = 2*α*_*a*_ + *α*_*c*_) are in good agreement with the previous direct theoretical calculations,^13,14^ and the results of all these theoretical calculations are in a qualitative agreement with experimental data.^13^ This finding reflects the accuracy and reliability of our calculated linear TECs. (iii) *α*_*c*_ is systematically and appreciably larger than *α*_*a*_. For example, at 300 K the calculated value of *α*_*c*_ (of 8.9 × 10^−6^ K^−1^) is about 60% larger than that of *α*_*a*_ (of 5.6 × 10^−6^ K^−1^).

As for the thermal expansion of the considered phases of BeF_2_, the features to note from Fig. 5 are the following. (i) Unlike ZnF_2_, the calculated values of both *α*_*a*_ and *α*_*c*_ are always positive for all of the considered phases of BeF_2_. (ii) Both *α*_*c*_ and *α*_*a*_ of the α-cristobalite structure are very close to those of α′-cristobalite, and hence only those of the former phase will be discussed below. (iii) In the considered *T*-range, *α*_*a*_(*T*) of the α-quartz structure is slightly larger than *α*_*c*_(*T*), whereas *α*_*a*_(*T*) of the α-cristobalite phase is much smaller than *α*_*c*_(*T*). (iv) The large *α*_*c*_ and *α*_*a*_ lead to very large *α*_v_ for both phases of BeF_2_. For example, at 300 K, the values of *α*_v_ are of 77.6 and 169.9 × 10^−6^ K^−1^ respectively for the above two phases of BeF_2_, compared to that of 20.0 × 10^−6^ K^−1^ for ZnF_2_. Our largest calculated linear TEC is of ∼95 × 10^−6^ K^−1^ at 300 K for *α*_*c*_ of the α-cristobalite phase. This is indeed large compared to the experimental linear TECs at 300 K of four fluorites, *i.e.*, CaF_2_, SrF_2_, BaF_2_, and PbF_2_ (ref. 41) that range between 18.1 and 29 × 10^−6^ K^−1^, but still is somewhat smaller than the measured linear TEC value of 163.9 × 10^−6^ K^−1^ of an Ti–Nb alloy.^42^

**Fig. 6:**
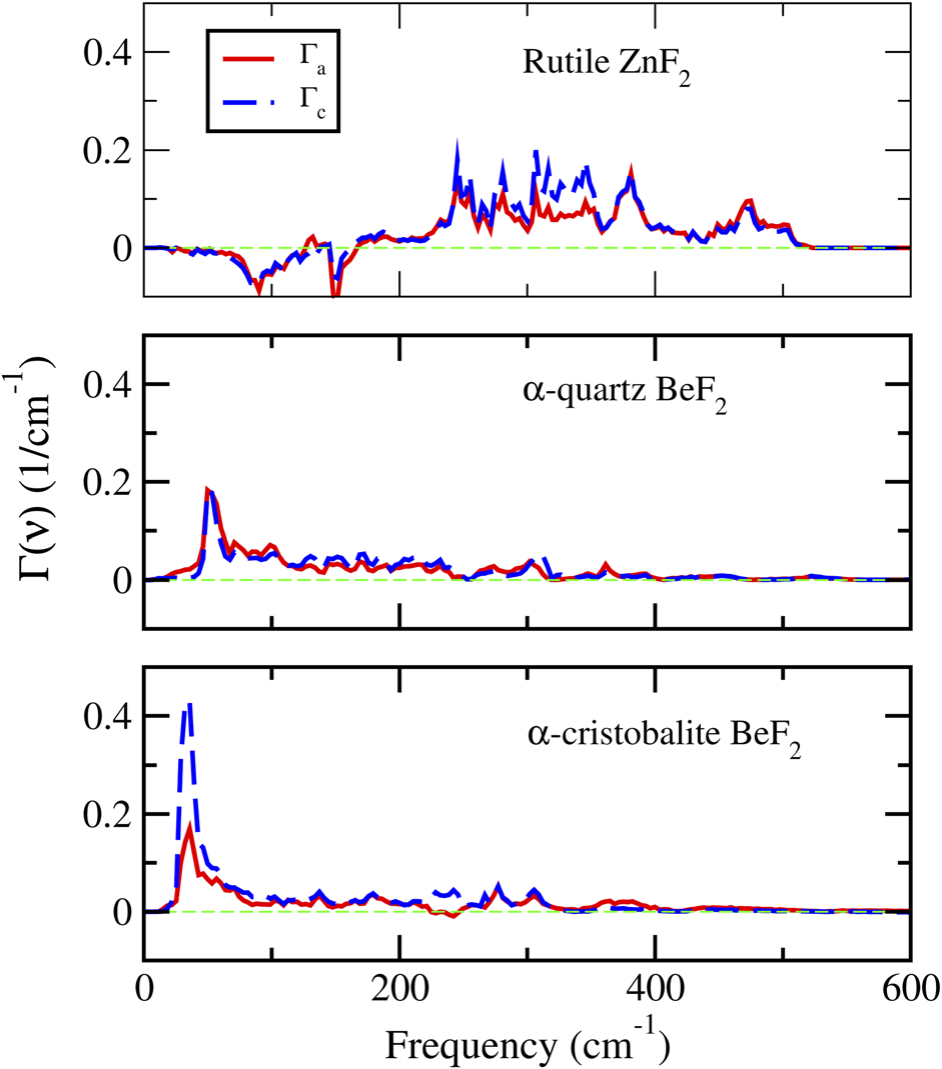
The PDOS weighted by the Grüneisen parameters of the rutile ZnF_2_, and the α-quartz and α-cristobalite phases of BeF_2_.

The key physically insightful quantity for the interpretation of the above results is the PDOS weighted by the Grüneisen parameters, *Γ*_*i*_(*ν*), defined in eqn (2). Fig. 6 shows *Γ*_*i*_(*ν*) of ZnF_2_, and the α-quartz and α-cristobalite phases of BeF_2_. The important features to note from this figure are the following. (i) For ZnF_2_, the low-frequency modes (*ν* < 150 cm^−1^) have negative Grüneisen parameters, which lead to negative *Γ*_*i*_(*ν*) in this *ν* range. Since low-frequency modes are easily thermally excited, this finding explains the observed NTE in ZnF_2_. Moreover, by inspecting the differences between *Γ*_*a*_(*ν*) and *Γ*_*c*_(*ν*) one can easily understand why *α*_*a*_ is always lower than *α*_*c*_. (ii) The *Γ*_*i*_(*ν*) of the considered phases of BeF_2_ are always positive, which reflects the dominance of positive mode Grüneisen parameters in these phases. This explains the lack of NTE in the considered phases of BeF_2_. (iii) The *Γ*_*a*_(*ν*) and *Γ*_*c*_(*ν*) of α-quartz BeF_2_ have comparable magnitudes, with *Γ*_*c*_(*ν*) being smaller than *Γ*_*a*_(*ν*) for *ν* < 100 cm^−1^, which explain the comparable magnitudes and ordering of its *α*_*c*_ and *α*_*a*_. (iv) The peak in *Γ*_*c*_(*ν*) of the α-cristobalite phase around *ν* ∼ 34 cm^−1^ is much higher than that of the *Γ*_*a*_(*ν*), which results in a large *α*_*c*_ compared to *α*_*a*_. This finding means that, in this *ν*-range, the positive mode Grüneisen parameters associated with the out-of-plane deformation are significantly larger than those associated with the in-plane deformation. The large Grüneisen parameters can be viewed as a manifestation of strong anharmonic effects in the α-cristobalite and α′-cristobalite structures of BeF_2_. However, it should be noted that large Grüneisen parameters are not the only factor that is responsible for the giant *α*_v_ of the α-cristobalite: the elastic property *via* the negative (and with a large magnitude) *C*_13_ elastic constant (see Table 2 and eqn (1)) plays also a major role. The above findings explain the much larger volumetric TEC of the α-cristobalite phase of BeF_2_, compared to that of the α-quartz phase, which, in turn, is larger than that of ZnF_2_.

## 4. Summary

First-principles calculations are performed to investigate the structural, elastic, and vibrational properties of the rutile structure of ZnF_2_ and three crystal structures of BeF_2_ (α-quartz, α-cristobalite and its similar phase with space group *P*4_3_2_1_2). The so-obtained phonon density of states, mode Grüneisen parameters, and elastic constants are used to study the linear thermal expansion coefficients (TECs) of the compounds mentioned above, within a Grüneisen formalism. We have used deformations that preserve the symmetry of the crystal to obtain the Grüneisen parameters. The considered crystal structures of both systems are found to be mechanically stable. The calculated physical quantities for both systems are in very good agreement with the available experimental data and previous theoretical results. For ZnF_2_, the calculated linear TECs, *α*_*a*_ and *α*_*c*_, along the *a* and *c* directions are consistent with the experimental *T*-variations of the corresponding lattice parameters, respectively. The volumetric TEC *α*_v_ computed from these linear TECs is in qualitative agreement with experiment at low temperatures, including negative thermal expansion (NTE) behavior. The considered phases of BeF_2_ are not NTE materials, and their linear TECs are much higher than those of ZnF_2_, especially for the α-cristobalite phase. The elastic constants, high-frequency dielectric constants, Born effective charge tensors, and TECs of the considered phases of BeF_2_ are reported in this work for the first time and could serve as predictions.

## Conflicts of interest

There are no conflicts to declare.

## Supplementary Material
